# Impact of UV Irradiation on the Chitosan Bioactivity for Biopesticide Applications

**DOI:** 10.3390/molecules28134954

**Published:** 2023-06-23

**Authors:** Solène Meynaud, Gaël Huet, Daphnée Brulé, Christian Gardrat, Benoit Poinssot, Véronique Coma

**Affiliations:** 1 Laboratoire de Chimie des Polymères Organiques, Université de Bordeaux, CNRS, Bordeaux INP, UMR 5629, 16 Avenue Pey-Berland, F-33600 Pessac, France; solene.meynaud@bordeaux-inp.fr (S.M.); gaelhuet@orange.fr (G.H.); christian.gardrat@u-bordeaux.fr (C.G.); 2 Agroécologie, CNRS, INRAE, Institut Agro, University Bourgogne, F-21000 Dijon, France; daphnee.brule@inrae.fr (D.B.); benoit.poinssot@u-bourgogne.fr (B.P.)

**Keywords:** chitosan, UV radiation, sunlight, bioactivity, eliciting properties, grapevine, MAPKs, downy mildew

## Abstract

Chitosan is known for its antimicrobial and antifungal properties that make it a promising candidate for plant protection. However, when sprayed in open fields, the bioactivity of chitosan significantly diminishes, suggesting a possible influence of sunlight on chitosan structure. This study aimed to investigate the effects of UV radiation, by using artificial UV sources simulating sunlight, on the stability of chitosan. A powdered chitosan with a low polymerization degree was selected and analyzed using various physicochemical methods, both before and after irradiation. Some minor differences appeared. UV spectra analysis revealed the disappearance of initially present chromophores and the emergence of a new band around 340 nm, potentially indicating the formation of carbonyl compounds. However, elemental analysis, MALDI-TOF spectra, polymerization degree, and infrared spectra did not exhibit any clear structural modifications of chitosan. Interestingly, irradiated powdered chitosan samples maintained their bioactivity, including their eliciting and antifungal properties. In the case of grapevine, irradiated chitosan demonstrated effectiveness in controlling grapevine diseases such as downy mildew, contradicting the assumption that sunlight is responsible for the decreased effectiveness of chitosan in open field conditions.

## 1. Introduction

**Figure 1:**
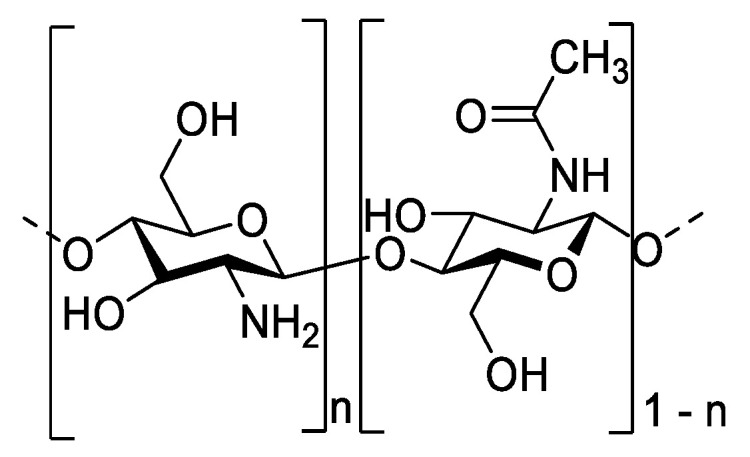
Chemical structure of chitosan with A− and D−units randomly distributed along the chain.

Chitosan, a natural polymer obtained through the deacetylation of chitin, is mainly extracted from the exoskeletons of crustaceans [1], the cuticles of insects [2] or from the cell walls of fungi [3]. It constitutes ß-(1→4)-D-glucosamine (D-units) linked to N-acetyl-D-glucosamine (A-units) with a variable degree of acetylation (Figure 1).

Chitosan has emerged as a potentially applicable polysaccharide in many fields ranging from food packaging [4] to biomedicine [5], pharmaceuticals [6], cosmetics [7], environment [8], and agriculture. In agriculture, chitosan offers a potential solution for reducing the utilization of chemicals and pesticides that contribute to soil pollution and pose health risks to living organisms [9,10]. Chitosan is authorized as a plant protection product according to the European Regulation EC 1107/2009 and Commission Implementing Regulation EU 2022/456 [11]. Chitosan has been widely studied as an efficient tool to trigger immune responses in plants [12,13]. In grapevine, chitosan elicits a variety of defense responses including the phosphorylation of mitogen-activated protein kinases (MAPKs), phytoalexin production, the expression of defense genes, and chitinase or glucanase activities. Together, this leads to resistance against the necrotrophic fungus *Botrytis cinerea*, the biotrophic fungus *Erysiphe necator*, and the obligate biotrophic oomycete *Plasmopara viticola*, the causal agents of grey mold, powdery mildew, and downy mildew, respectively [14,15,16]. Spraying aqueous chitosan solutions could lead, after the evaporation of water, to the formation of a thin film coat on the plant’s surface [17]. However, the effectiveness of plant protection achieved using chitosan is considerably higher in laboratory and greenhouse settings compared to open fields [18]. The reasons for this disparity remain unclear, but two primary hypotheses can be proposed: (i) lixiviation caused by rainfall and morning dew, and/or (ii) the degradation of chitosan due to sunlight exposure in the presence of air, specifically the photo-oxidation of chitosan. This paper focuses on investigating the second hypothesis.

The literature has extensively discussed the impact of photo-irradiation techniques on various biopolymers, including chitosan [19]. Several studies have described the photodegradation of chitosan, both in film form and in solution, in the presence of air. These investigations have utilized UVB irradiation, typically at approximately 254 nm [20,21,22,23,24,25]. More recently, chitosan films were irradiated using UVA at a wavelength higher than 300 nm [26]. The pairing of UV light and hydrogen peroxide has also been studied [27]. Blends of chitosan with other polymers such as poly(vinylpyrrolidone), poly(ethylene oxide), and pectin [28,29,30] as well as chitosan modified with keratin, silk fibroin, and tannic acid [31,32,33] were also irradiated at 254 nm and studied. A recent study focused on examining the photo-oxidation of chitosan in the presence of citric acid, specifically at a wavelength of 340 nm [34]. The main chemical results of UV irradiation indicated a decrease in the molecular weight of chitosan as a result of β-D-(1→4) glycosidic bond scissions and the formation of carbonyl compounds.

In this paper, we aim to investigate the impact of ultraviolet light on a solid chitosan with a low polymerization degree in the presence of air. The chitosan samples used in this study were subjected to irradiation in their as-received state and in their solid acetate form, simulating the chitosan present on leaves after pulverization from an acetic acid formulation. Both irradiated and non-irradiated chitosan samples underwent analysis using various physicochemical methods. Furthermore, the biological properties of these chitosans were investigated, specifically in terms of their elicitation potential and effectiveness in controlling grapevine diseases, such as downy mildew.

## 2. Results

The solid chitosan was irradiated as received by the supplier and in its solid acetate form. Two irradiation methods at about 340–350 nm were used, which differ through their irradiance value.

### 2.1. Characterization of Non-Irradiated and Irradiated Chitosans

#### 2.1.1. Physicochemical Characteristics

The following characteristics of the samples (polymerization degree, X-ray photoelectron spectrometry, elemental analysis, deacetylation degree) were studied, and the results are presented in Table 1.

To determine the deacetylation degree (DD) of chitosan using elemental analysis, it is necessary to ascertain the weight percentage ratio of carbon to nitrogen (C/N) [35]. Additionally, it is crucial to ensure that the samples do not contain nitrogen-containing impurities such as proteins [36]. Furthermore, the presence of water within the chitosan chains does not affect these analyses, as the percentages of oxygen and hydrogen have no influence on the C/N ratio. Therefore, the hydrogen percentage values were not considered in the analysis.

X-ray photoelectron spectroscopy (XPS) was employed to measure the elemental composition of the atoms on the surface of the sample. A typical XPS survey of chitosan revealed the presence of the expected elements, namely, carbon (C), nitrogen (N), and oxygen (O). Additionally, minor traces of calcium (Ca), silicon (Si), and/or chlorine (Cl) may be detected. These components can be attributed to the processing of crustacean exoskeletons, which serve as the source material for chitosan production [37]. Nevertheless, a high proportion of chlorine indicated that the commercial chitosan is in its chlorhydrate form.

The polymerization degree (DP) calculated from ^1^H NMR measurements remained constant and was not affected by the irradiation time (Table 2).

Finally, no significant differences were observed between non-irradiated and irradiated samples in terms of acetylation degree calculated from elemental analysis, atomic composition of the surface, and polymerization degree of the chitosan.

#### 2.1.2. Spectral Characteristics

- Fourier-Transform Infrared Spectroscopy

**Figure 2:**
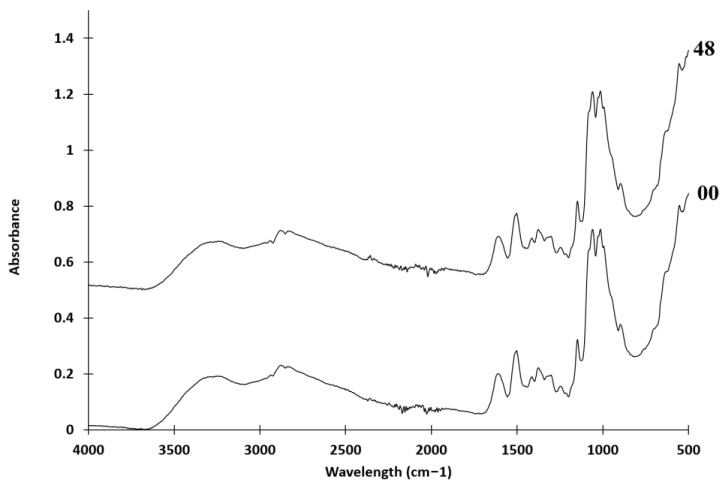
FTIR spectra of powdered non-irradiated (00) and chitosan irradiated for 48 h (48) using a UV-box.

FTIR is a valuable tool for investigating structural modifications in chitosan. Infrared spectra are shown in Figure 2. Comparing the spectra before and after irradiation, recovered ν_O-H_ and ν_N-H_ bands were observed between 3100 and 3500 cm^−1^, glycosidic ν_C-O-C_ bands were observed between 850 and 1150 cm^−1^, and the stretching vibration ν_C = O_ (amide I) appeared at 1616 cm^−1^, while δ_NH3+_ appeared at 1510 cm^−1^ [38]. As a result, no noticeable impact of irradiation on the infrared spectra was observed.

- Mass spectrometry

**Figure 3:**
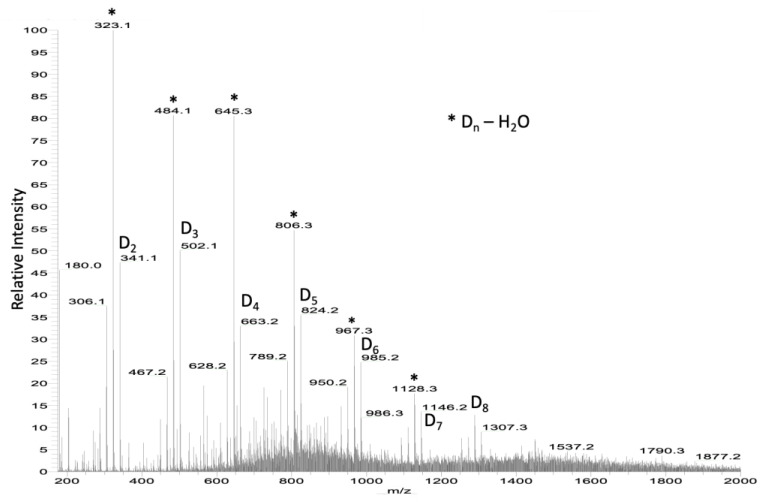
ESI mass spectrum of chitosan. * D_n_-H_2_O, means dehydrated D_n_.

Chitosan was studied using mass spectrometry, electrospray ionization (ESI), and matrix-assisted laser desorption/ionization time-of-flight mass spectrometry [39,40]. Using ESI, ions with deacetylated D-type structures were identified from D_2_ to D_6_ accompanied by their dehydrated ions in the spectra of chitosan samples (Figure 3).

Using collision-induced dissociation, it was demonstrated that the dehydrated ions came from the deacetylated ions through the loss of water. Consequently, it was proposed that the abundance of a global structure, denoted as D_n_, was actually the combined result of the abundance of D_n_ and the (D_n_-H_2_O) peaks. A comparison was conducted between the electrospray ionization (ESI) spectra of non-irradiated and irradiated chitosan samples exposed to UV-box irradiation for 7 days and Q-Sun XE-1 irradiation for 4 days, respectively (as shown in Table 3). Notably, after chitosan irradiation, there appeared to be an observable increase in the abundances of chitosan oligosaccharides (COS).

In the MALDI-TOF spectrum of non-irradiated chitosan, some deacetylated ions of the type D_n_A_1_ were identified when using a reflectron (spectrum not shown). Unfortunately, for unknown reasons, the results were not reproducible in the spectra of the irradiated chitosan. Therefore, it was not possible to determine whether there was a slight deacetylation of the chitosan under UV light.

- Comparison of UV spectra of non-irradiated and irradiated chitosans in UV-box.

**Figure 4:**
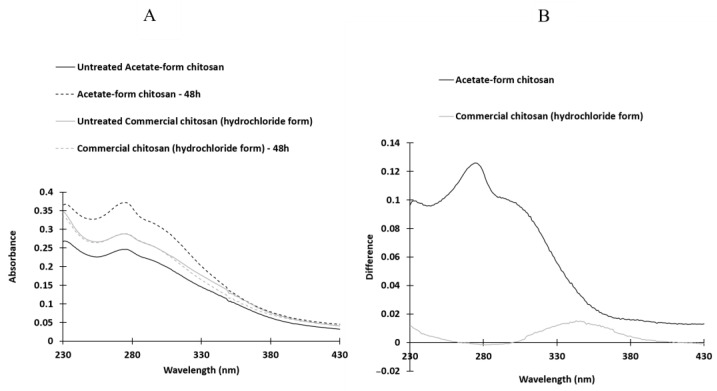
Comparison of UV spectra of non-irradiated and irradiated chitosan samples over a period of 48 h using a UV-box (**A**) and absorbance difference spectra (**B**). The spectra were recorded in acetic acid as the solvent. The chitosan samples were irradiated in their acetate form (represented by the black line) or hydrochloride form (represented by the grey line).

Ultraviolet spectra were recorded using chitosan dissolved in acetic acid (Figure 4). The spectra showed similarities, but there were noticeable differences in absorbance between non-irradiated and irradiated chitosan samples. In the case of solid acetate samples, a shoulder appeared around 305 nm, while in solid hydrochloride chitosan, a new band emerged around 340 nm. Additionally, some chromophores near 280 nm disappeared in the irradiated solid hydrochloride chitosan samples.

**Figure 5:**
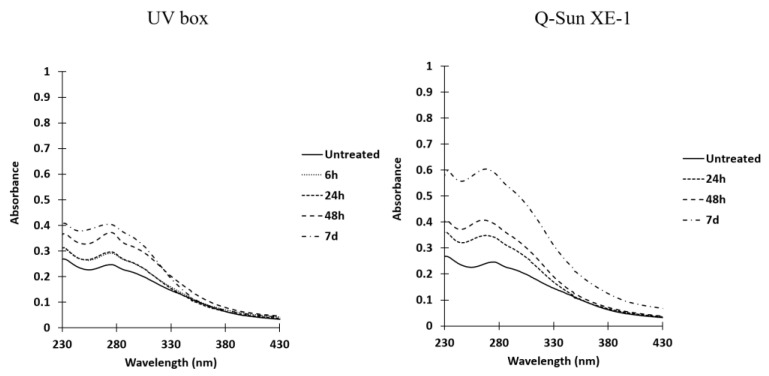
UV spectra of non-irradiated and irradiated chitosan solubilized in acetic acid using a UV-box or a Q-Sun XE-1 system.

- Comparison of UV spectra between non-irradiated and irradiated chitosan as a function of irradiation time (Figure 5).

The solid chitosan acetate exhibited a slight absorption peak near 310 nm in the ultraviolet spectra, which intensified with longer irradiation times. This absorbance was observed with both of the irradiation devices utilized, but it was more pronounced when the Q-Sun XE-1 system was employed. The comparison of absorbance differences between non-irradiated and irradiated acetate samples revealed the emergence of a shoulder between 280 and 330 nm (spectra not shown).

### 2.2. Bioactivity of Non-Irradiated and Irradiated Chitosan

**Figure 6:**
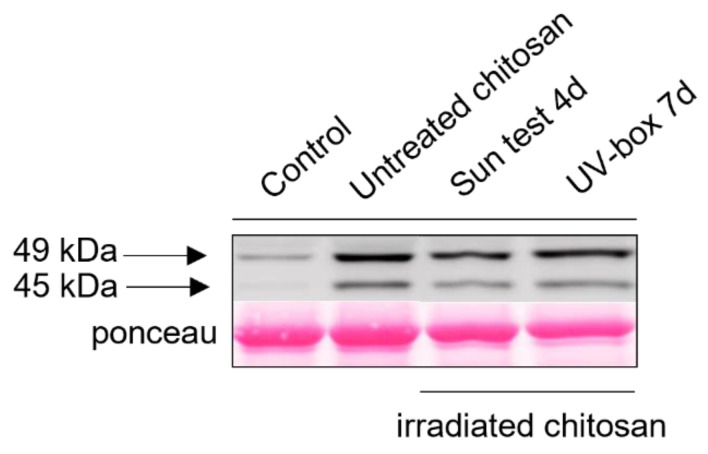
Irradiated-chitosan-induced phosphorylation of MAPKs in grapevine. Activation of two MAPKs detected using immunoblotting with an antibody raised against the human phosphorylated extracellular regulated protein kinase ½ (α-pERK1/2) in grapevine leaf discs treated with non-irradiated or Sun test (Q-Sun XE-1 system)- or UV-box-irradiated chitosan (1 mg/mL) or water (negative control). Homogeneous loading was checked using Ponceau red staining.

To determine the influence of UV on the chitosan’s abilities to induce defense responses and resistance in grapevine, we first investigated an early signaling event such as MAPKs phosphorylation. Non-irradiated and irradiated chitosan under its acetate form induced a rapid phosphorylation of two MAPKs with relative molecular masses of 45 and 49 kDa, which was not observed in water-treated control leaf discs. This activation of MAPKs seems to be similar between non-irradiated and irradiated chitosan and does not differ regardless of the method of irradiation (Figure 6).

**Figure 7:**
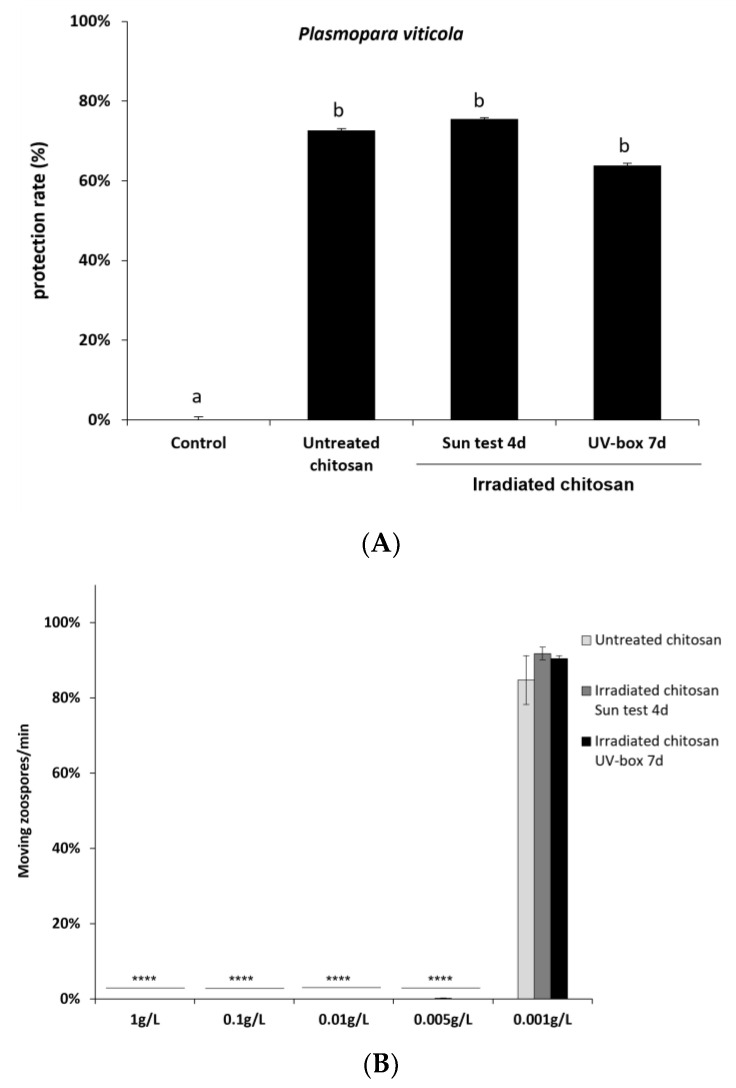
Irradiated-chitosan-induced resistance in grapevine and its toxicity effects on *Plasmopara viticola.* (**A**) Grapevine cuttings were sprayed with non-irradiated and irradiated chitosan (15 mg/L) 48 h before inoculation. Leaf discs were cut 5 dpi and the disease caused by *P. viticola* was assessed at 7 dpi. The sporulating leaf area was evaluated using image analysis Visilog 6.9 software [41]. Values represent the mean of protection rate ± SE (n = 36 discs from three different plants/conditions) from three independent biological experiments. Different letters indicate a statistically significant difference between treatments (Kruskal–Wallis followed by Mann–Whitney post hoc with *p* < 0 05). (**B**) Toxicity effects on the motility of *Plasmopara viticola* zoospores. *P. viticola* sporangia were treated with increasing concentrations of non-irradiated and irradiated chitosan, and released zoospores moving on a 1 mm^2^ square of a Malassez hemocytometer were counted for one minute. Values represent the mean ± SE (n = 9) of three independent experiments and are expressed as a percentage of the control set as 100%. Asterisks indicate significant differences relative to the control using an unpaired heteroscedastic Student’s *t* test; ****, *p* < 0.0001.

To further characterize the immune responses triggered by irradiated chitosan, we also investigated its ability to induce resistance against downy mildew in grapevine. Leaves were treated with non-irradiated or irradiated chitosan 48 h prior to inoculation with the biotrophic oomycete *P. viticola*. Non-irradiated and irradiated chitosan treatment significantly reduced *P. viticola* sporulation, and there was no significant difference of protection efficiency between non-irradiated and irradiated chitosan (Figure 7A).

The toxicity of irradiated chitosan was then assessed on *P. viticola* by counting the number of moving zoospores after chitosan or water treatment. As with non-irradiated chitosan, irradiated chitosan remains toxic at very low concentrations on *P. viticola* zoospores. Sporangia treated with non-irradiated and irradiated chitosan at concentrations from 1 to 0.005 g/L did not release any moving zoospores (Figure 7B). At 0.001 g/L, there are as many moving zoospores as in the control. Taken together, these results suggest that UV irradiation of chitosan has no impact on its eliciting and antifungal activities and highlights a strong direct biocide effect of non-irradiated and irradiated chitosan.

## 3. Discussion

As mentioned in the introduction, the use of chitosan as a biopesticide in the field does not yield the expected results, despite the promising bioactivities observed in laboratory or greenhouse settings. Several hypotheses can be proposed, one of which relates to the potential influence of solar light on the structure of chitosan. Conducting experiments in the open field under controlled conditions is challenging due to the rapidly changing weather conditions throughout the day. Therefore, in this work, laboratory experiments were carried out using chitosan with a low degree of polymerization (due to its significant bioactive properties) and dedicated devices for continuous irradiation using ultraviolet lamps to simulate solar radiation. It is important to note that in-depth studies on powdered chitosan have been limited. The chosen chitosan in this study does not form a film and is present only in its powdered form after application via spraying on plants.

Two artificial sources of UV radiation were employed to simulate the ultraviolet contribution of sunlight, primarily at a wavelength range of 340–350 nm. The objective was to assess the potential structural degradation of chitosan and its impact on its biological properties, specifically its eliciting and antifungal activities.

In the UV spectra of solid chitosan in its hydrochloride or acetate form, a band was observed between approximately 280 and 430 nm. This phenomenon has been previously noted by various authors, including Yu et al., in the case of acetate chitosan solutions [42]. To the best of our knowledge, the exact origin of this broad absorption band has not been definitively identified. After irradiating the solid chitosan, the UV spectra exhibited the disappearance of initially present chromophores around 280 nm, and the emergence of a new band around 340 nm for the hydrochloride form and the formation of a band at approximately 300 nm for the acetate form.

The intensity of the new band increased with prolonged irradiation time, although these modifications appeared to be less pronounced in the hydrochloride form. However, according to El-Sawy et al. [43], who conducted irradiation on chitosan under ^60^Co (γ rays), this absorption band may be attributed to the n→δ* transition of chitosan’s amino groups or the n→π* transition of carbonyl or carboxylic groups. Under severe UV conditions (accelerated photo-oxidation at 60 °C), Bussière et al. [26] also suggested that this band could be attributed to the formation of carbonyl groups and the scission of glycosidic bonds. However, in our experimental conditions, it was not possible to reveal any evidence of such degradation in solid chitosan. The FTIR spectra indicated no significant differences between the chemical structures of irradiated and non-irradiated powdered chitosan. No new bands appeared or disappeared. Consequently, it can be concluded that there were no significant chemical transformations in the overall structure of the biomacromolecules. The primary possibility would be the cleavage of ß-D-(1→4) glycosidic bonds. Nevertheless, if such cleavages occurred, other physicochemical modifications should manifest, such as a reduction in the average polymerization degree and a substantial increase in low D_n_ fragments in ESI mass spectra. In reality, the former was not observed, and the latter was not particularly significant.

After the irradiation of solid chitosans, the UV spectra exhibited the disappearance of chromophores that were originally present in the non-irradiated samples. One possible hypothesis is the photodegradation of amide functions under irradiation. However, if this happened, a change in the carbon content of the sample should be observed, which is not the case.

Consequently, the various physicochemical analyses performed did not reveal any noticeable structural modifications resulting from chitosan irradiation. Additionally, it was found that irradiation had no impact on the elicitation of antifungal activities.

In our specific experimental conditions, it appeared that the UV irradiation did not have sufficient energy to significantly alter the powdered low-molecular-weight chitosan, in accordance with Pandit et al. stating that UV treatment alone is ineffective in degrading chitosan [19]. Additionally, it should be noted that the irradiation procedure employed in this study did not accurately replicate the solar spectrum but rather imposed more rigorous conditions [44].

Consequently, two conclusions can be drawn: Firstly, in the conditions utilized in this study, the UV irradiation alone, which simulates a 5-day exposure in the field, is unlikely to induce substantial modifications in the chitosan structure, as previously observed in the literature [22], and chitosan eliciting and anti-downy mildew properties. Secondly, the lower effectiveness of chitosan in open field applications would not be attributed to sunlight exposure.

## 4. Materials and Methods

### 4.1. Materials

The chitosan used in this study was obtained from Elicityl (Crolles, France) through the process of the acidic hydrolysis of chitosan from crustacean shells. The chitosan was stored at room temperature, away from sunlight, to maintain its integrity. Its main characteristics, including its physical and chemical properties, are provided in Table 1 *Before irradiation*. Prior to the bioactivity assays, the chitosan was dissolved in ultrapure water. Glacial acetic acid was supplied by Fisher Chemical (United Kingdom).

Grapevine (*V. vinifera* cv. Marselan) cuttings were grown in a greenhouse until they had developed 6–8 leaves. The first and second youngest adult leaves from each plant were used for experiments. Grapevine downy mildew (*P. viticola*) was routinely maintained on *Vitis vinifera* cv. Marselan plants as previously described [45].

### 4.2. Methods

#### 4.2.1. Elemental Analyses

Elemental analyses were performed on a ThermoFisher FlashEA-1112 microanalyzer equipped with a sampler changer. Samples were prepared by being weighed precisely (approximatively 1.5 mg) on a tin capsule with a Mettler Toledo XPR2U microbalance. The tin capsules were then compressed into a small cube. In the analyzer, the samples underwent dynamic “flash” combustion at 930 °C. Nitrogen, carbon, and hydrogen were then recombined using a series of reduction and oxidation reactions to give dinitrogen, carbon dioxide, and water. These gases were then separated on a chromatographic column, detected by a katharometer, and then quantified by the integration of a peak resulting from the variation in the thermal conductivity of the gases, leading to the percentages of C, H, and N.

The deacetylation degree was calculated with the C/N weight percentage ratio according to Dos Santos et al. [46].

#### 4.2.2. Degree of Polymerization (DP) by ^1^H NMR

The measurements of average DPs are based on the integration of the signals corresponding to protons belonging to the non-reducing ends of the polymer chains and those belonging to the reducing ends as described in the literature [47]. ^1^H-NMR spectra were registered at room temperature using a Liquid-state 400 MHz NMR spectrometer (Bruker ADVANCE I) with 32 scans. Samples were prepared with 20 mg of chitosan and mixed with 1 mL of D_2_O and 10 µL of DCl (7.4 M).

#### 4.2.3. Analysis of Chitosan Powder Surface using XPS

A ThermoFisher Scientific K-ALPHA spectrometer was used for XPS surface analysis with a monochromatized Al-Kα source (hν = 1486.6 eV) and a 400 μm X-ray spot size. Powders were pressed onto indium foils. The full spectra (0–1100 eV) were obtained with a constant pass energy of 200 eV, while high-resolution spectra were recorded with a constant pass energy of 40 eV. Charge neutralization was applied during the analysis. High-resolution spectra were quantified using Avantage software provided by ThermoFisher Scientific (Waltham, MA, USA).

#### 4.2.4. Mass Spectrometry

- Electrospray

Electrospray analyses were performed on a linear trap quadrupole (LTQ) mass spectrometer (Thermo Fisher Scientific, Waltham, MA, USA) in positive ion mode using direct infusion of the samples in a mixture of water/methanol (4/1, *v*/*v*) (0.1 mg/mL). Electrospray source parameters were as follows: capillary voltage +20 V, tube lens voltage +90 V, capillary temperature 300 °C, sheath and auxiliary gas flow (N^2^) 8 and 5, sweep gas 0, spray voltage 3.6. Spectra were acquired by full range acquisition covering *m/z* 50–2000. Collision-induced dissociations were made with helium.

- MALDI-TOF

MALDI-TOF spectra were registered on a Voyager mass spectrometer (Applied Biosystems, Waltham, MA, USA) equipped with a pulsed nitrogen laser (337 nm) and a time-delayed extracted ion source. Spectra were recorded in positive ion mode using reflectron mode with an accelerating voltage of 20 kV. The chitosan samples were dissolved in a mixture made of H_2_O/MeOH (50/50 *v*/*v*) and acetic acid (0.1%, *v*/*v*) at 10 mg/mL. The 2,5-dihydroxybenzoic acid (DHB) matrix solution was prepared by dissolving DHB (10 mg) in MeOH (1 mL). A MeOH solution of cationization agent (NaI, 10 mg/mL) was also prepared. The solutions were combined in a 10:1:1 volume ratio of matrix to sample to cationization agent. One to two microliters of the obtained solution were deposited onto the sample target and vacuum dried.

#### 4.2.5. ATR-FTIR Spectra

Infrared spectra were registered using a FTIR spectrometer with a diamond crystal (PIKE technologies/Gladi Atrvertex 70, Bruker, France) from 4000 to 400 cm^−1^ (32 scans, resolution 4 cm^−1^).

#### 4.2.6. Ultraviolet Irradiation

Solid commercial chitosan was irradiated as received from the supplier and under its acetate solid form in Petri dishes.

- Preparation of chitosan acetate from commercial chitosan

To prepare the powdered chitosan, 300 mg of it was dissolved in 10 mL of 1% (*v*/*v*) acetic acid and stirred until complete solubilization. The resulting solutions were then transferred into Petri dishes (diameter of 8 cm). Subsequently, the samples were dried at 40 °C for 48 h prior to conducting the UV irradiation tests. The dried powder was evenly spread across the surface of the Petri dishes.

- UV irradiation of solid commercial chitosan and chitosan acetate

Two different methods were used to irradiate the chitosan samples:

(1) The Petri dishes were placed in a laboratory-made irradiation chamber (named “UV-box” in the text) equipped with four black light UV lamps (Mazdafluor TFWN 18) emitting mainly at 350 nm. The distance between the lamp and the sample was ~25 cm, the light intensity was 0.25 mW/cm^2^, and the temperature was maintained at 25 °C using a fan.
(2) The Petri dishes were covered with a borosilicate lid to protect the Q-Sun XE-1 test chamber (Q-Lab Corporation, Westlake, OH, USA) from potential gas and dust that could go outside. The device was equipped with a xenon source and a Daylight-Q filter. The irradiance was equal to 0.47 W/m^2^ at 340 nm. The chamber was ventilated to maintain the temperature at 50 °C.

The irradiation times were fixed at different values for the UV-box and the Q-Sun XE-1.

- UV–vis analysis of irradiated chitosans

After irradiation, the powdered chitosan samples were dissolved in an aqueous solution of acetic acid (1% *v*/*v*) in order to obtain a concentration of 5 mg/mL. Spectra were recorded on a UV–visible spectrophotometer (Agilent Cary 100) from 250 nm to 430 nm using quartz cells (length 1 cm).

#### 4.2.7. MAPK Activation

Discs of grapevine leaves from greenhouse cuttings were pre-infiltrated with ultrapure water then equilibrated, with the abaxial face on ultrapure water, for 4 h in a 6-well plate. They were then treated by substituting the water with non-irradiated or irradiated chitosan (1 mg/mL) or water (negative control) and harvested 20 min post-treatment. MAPKs activation was detected after immunoblotting of the extracted proteins (20 µg) using an anti-p42/44-phospho-ERK antibody (Cell Signaling). The revealing step was performed on an Amersham™ ImageQuant™800 (Cytiva) using ECL™ Prime as a Western blotting detection reagent. Transfer quality and homogeneous loading were checked using Ponceau red staining. Three independent experiments were performed.

#### 4.2.8. Downy Mildew Assay

For *P. viticola* infection on grapevine cuttings, the lower leaf surface was sprayed with non-irradiated or irradiated chitosan (15 µg/mL) or water (control). Two days post-treatment (dpt), treated leaves were sprayed with a freshly prepared suspension of sporangia (2 · 10^4^ sp/mL) and plants were maintained in 100% humidity for 4 h. Leaf discs were cut 5 days post-inoculation (dpi) and transferred on moist Whatman paper in a plastic box maintained in 100% humidity under a 10/14 h day/night cycle at 20/18 °C. Infection intensity was assessed as 7 dpi by measuring the sporulating area by using image analysis Visilog 6.9 software [41].

For toxicity assays on *P. viticola* zoospores, a suspension of *P. viticola* sporangia (1 · 10^5^ sp/mL) prepared in ultrapure water was treated with increasing concentrations of non-irradiated or irradiated chitosan (0.001, 0.005, 0.01, 0.1, and 1 mg/mL). Two hours later, released zoospores moving on a 1 mm^2^ square of a Malassez hemocytometer were counted for one minute.

## 5. Conclusions

The study aimed to assess the influence of sunlight on the stability and bioactivity of powdered chitosan with a low polymerization degree. To simulate sunlight exposure, artificial UV sources were utilized. A range of physico-chemical methods, including elemental analysis, NMR to calculate the polymerization degree, MALDI-TOF, IR, XPS, and UV spectroscopy, were employed to compare the characteristics of chitosan before and after irradiation. Although minor differences were observed in the UV spectra, no significant structural modifications of chitosan were detected under the experimental conditions. Additionally, the anti-downey mildew and eliciting properties of chitosan were unaffected by UV irradiation. These findings suggest that the reduced effectiveness of chitosan in open field conditions cannot be solely attributed to sunlight exposure.

## Figures and Tables

**Table 1:** Characteristics of the selected chitosan in terms of its degree of polymerization (DP), X-ray photoelectron spectroscopy (XPS), elemental analysis, and deacetylation degree (DD) both before and after 48 h of irradiation in a UV-box.

Chitosan Sample	DP ^1^	XPS Atomic %						Elemental Analysis ^2^		DD %
		C_1s_	O_1s_	N_1s_	Ca_2p_	Si_2p_	Cl_2p_	%C	%N	
Before irradiation	9	54.32	31.86	7.03	0.24	0.40	6.16	32.46 ± 0.01	5.97 ± 0.01	83 ± 1
After irradiation	8	53.62	32.42	7.29	0.19	0.43	6.06	32.39 ± 0.04	5.95 ± 0.01	82 ± 1

^1^—See the evolution of the DP in Table 2; ^2^—Means and standard deviations calculated from three repetitions.

**Table 2:** Evolution of the polymerization degree vs. the irradiation time.

Irradiation Time (h)	UV-Box DP	Q-Sun XE-1 DP
0	9	9
4	nd *	8
6	9	nd *
24	9	8
48	9	8
96	nd *	8
168	9	nd *

nd *—Not determined.

**Table 3:** Comparison of the relative abundances of D_n_ in non-irradiated and irradiated chitosan. The values presented include uncertainties from three repetitions.

D_n_	Non-Irradiated Chitosan	Irradiation Over 7 Days UV-Box	Irradiation Over 4 Days Q-Sun XE-1
D_2_	1000	1000	1000
D_3_	865 ± 2	1034 ± 2	1065 ± 2
D_4_	704 ± 5	909 ± 3	900 ± 2
D_5_	617 ± 7	689 ± 6	735 ± 4
D_6_	382 ± 10	371 ± 9	422 ± 9

## Data Availability

Not applicable.
